# Mutation in *PHACTR1* associated with multifocal epilepsy with infantile spasms and hypsarrhythmia

**DOI:** 10.1111/cge.13926

**Published:** 2021-01-27

**Authors:** Andrey V. Marakhonov, Magdalena Přechová, Fedor A. Konovalov, Alexandra Yu. Filatova, Maria A. Zamkova, Ilya V. Kanivets, Vladimir G. Solonichenko, Natalia A. Semenova, Rena A. Zinchenko, Richard Treisman, Mikhail Yu. Skoblov

**Affiliations:** ^1^ Laboratory of Genetic Epidemiology, Laboratory of Functional Genomics, Department of Genetic Counseling Research Centre for Medical Genetics Moscow Russia; ^2^ Laboratory of Integrative Biology, Institute of Molecular Genetics of the Czech Academy of Sciences Prague Czech Republic; ^3^ Signalling and Transcription Laboratory Francis Crick Institute London UK; ^4^ Independent Clinical Bioinformatics Laboratory Moscow Russia; ^5^ Laboratory of Regulatory Mechanisms in Immunity Institute of Carcinogenesis, N.N. Blokhin Russian Cancer Research Center Moscow Russia; ^6^ Laboratory of Molecular Pathology Genomed Ltd. Moscow Russia; ^7^ Medical Genetic Centre Filatov Moscow Pediatric Clinical Hospital Moscow Russia; ^8^ N.A. Semashko National Research Institute of Public Health Moscow Russia

**Keywords:** hypsarrhythmia, malignant migrating partial seizures of infancy, multifocal epilepsy with infantile spasms, PHACTR1, PP1

## Abstract

A young boy with multifocal epilepsy with infantile spasms and hypsarrhythmia with minimal organic lesions of brain structures underwent DNA diagnosis using whole‐exome sequencing. A heterozygous amino‐acid substitution p.L519R in a *PHACTR1* gene was identified. PHACTR1 belongs to a protein family of G‐actin binding protein phosphatase 1 (PP1) cofactors and was not previously associated with a human disease. The missense single nucleotide variant in the proband was shown to occur *de novo* in the paternal allele. The mutation was shown *in vitro* to reduce the affinity of PHACTR1 for G‐actin, and to increase its propensity to form complexes with the catalytic subunit of PP1. These properties are associated with altered subcellular localization of PHACTR1 and increased ability to induce cytoskeletal rearrangements. Although the molecular role of the PHACTR1 in neuronal excitability and differentiation remains to be defined, PHACTR1 has been previously shown to be involved in Slack channelopathy pathogenesis, consistent with our findings. We conclude that this activating mutation in PHACTR1 causes a severe type of sporadic multifocal epilepsy in the patient.

## Graphical abstract

Activating nature of the PHACTR1 L519R mutation. Left, PHACTR1 binds multiple G‐actin molecules (shown symbolically by a single red circle) which antagonise formation of the PHACTR1/PP1 complex, which will therefore form only when local G‐actin concentration falls below a threshold. Right, PHACTR1 L519R (star) exhibits reduced G‐actin binding affinity, allowing the PHACTR1/PP1 complex to form at higher G‐actin concentation. The L519R mutation affects PHACTR1 subcellular localisation as well, and it is therefore possible that its phenotype additionally reflects redistribution of PP1.

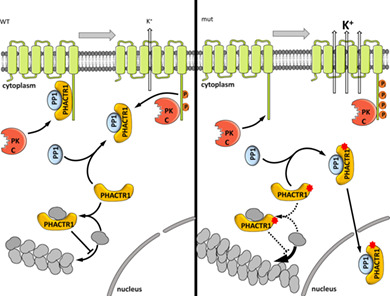

## 1 INTRODUCTION

Epilepsy is a diverse group of disorders characterized by recurrent seizures. The idiopathic form of epilepsy is thought to be genetically determined,^1^ and over 500 genes have been associated with epilepsy in human and mouse.^2^ Most cases are associated with mutations in genes encoding subunits of ion channels, neurotransmitter receptors or synaptic vesicle cycle proteins that play essential roles in neuronal activity.^3^ Nevertheless, these findings do not explain all cases of congenital forms of epilepsy, and new genes continued to be uncovered.^4^

Protein phosphatases and protein kinases have a crucial role in the functioning of the mammalian brain. The coordinated activity underlies two major forms of synaptic plasticity known as long‐term potentiation and long‐term depression (LTD).^5^, ^6^ LTD‐inducing stimuli promote distribution of PP1 to dendritic spines,^7^ where its association with the actin‐rich postsynaptic density (PSD) is critical for effective dephosphorylation of its substrates and down‐regulation of synaptic function.^8^, ^9^, ^10^ Numerous pathogenic conditions involve altered function of PP1 in the brain.^11^, ^12^, ^13^, ^14^ Epilepsy can also be regarded as exhibiting synaptic plasticity with elevated excitatory synapse activity.^15^ Modulation of kinase/phosphatase balance has been shown to induce status epilepticus and/or seizure activity in rats.^16^, ^17^

Two members of the Phactr family of G‐actin binding PP1 cofactors, PHACTR1 and PHACTR3, are highly expressed in the brain.^18^, ^19^ Interaction with PP1 appears critical for the function of PHACTR proteins.^20^, ^21^ The presence of PHACTR1 in the PSD indicates that it is likely to play a role in neuronal function,^18^ while PHACTR3 was shown to inhibit axon elongation in primary rat cortical neurons via regulation of actin dynamics.^19^ Moreover, Phactr3 was recently shown to be necessary for regulation of neuronal morphology by increasing the percentage of mature dendritic spines as well as up‐regulating dendritic complexity.^22^

Phactr family proteins interact with G‐actin through four conserved RPEL motifs (named after ultraconservative amino‐acids building up the motif).^18^, ^23^, ^24^, ^25^, ^26^ Studies in non‐neuronal cells have shown that PHACTR1/G‐actin interaction controls its subcellular localization and inhibits its interaction with PP1.^23^, ^24^

A recent study has demonstrated that PHACTR1 specifically interacts with Slack, a Na^+^‐activated K^+^ channel (K_Na_1.1) which is encoded by *KCNT1* gene, and dissociates from it upon channel activation.^27^ Mutations in Slack are associated with malignant migrating partial seizures of infancy (MMPSI), characterized by infantile seizures and intellectual disability.^28^ Interestingly, MMPSI mutations in Slack both constitutively activate the channel, and affect its interaction with PHACTR1, raising the possibility that the MMPSI phenotype may in part reflect altered PHACTR1 activity. Indeed, recently, it was demonstrated that *de novo* mutations in *PHACTR1* gene are associated with West syndrome characterized by infantile spasms, hypsarrhythmia, and mental retardation.^29^ Although authors do provide evidence for the pathogenic role of these *PHACTR1* mutations in a rodent model how this relates to pathogenesis of West syndrome remained unclear.

Here we used whole‐exome sequencing (WES) to identify a novel mutation in *PHACTR1* that occurred *de novo* in a proband with multifocal epilepsy with infantile spasms and hypsarrhythmia. We show that this mutation, L519R, reduces the affinity of PHACTR1 for G‐actin and increases its propensity to form complexes with the catalytic subunit of PP1 *in vitro*. These properties are associated with altered subcellular localization of PHACTR1 and increased ability to induce cytoskeletal rearrangements *in vivo*. We propose that L519R acts as an activating mutation of PHACTR1. Overall, our study provides a clue to the molecular pathogenesis of *PHACTR1*‐associated epileptic encephalopathy of infancy.

## 2 MATERIALS AND METHODS

### 2.1 Source of DNA

Peripheral blood samples were collected from affected individual and his parents as well as from 120 unrelated healthy control individuals of the Central Russian origin. Genomic DNA from the samples was extracted using standard protocols.

### 2.2 Editorial policies and ethical considerations

The clinical and molecular genetic study was performed in accordance with the Declaration of Helsinki and was approved by the Institutional Review Boards of the National Research Center for Preventive Medicine and Research Centre for Medical Genetics, Moscow, Russia, with written informed consent obtained from each participant and/or their legal representative, as appropriate.

### 2.3 Genetic analysis of the epilepsy‐affected family

#### 2.3.1 Whole exome sequencing

Whole exome sequencing in a proband was done by a commercial laboratory on Illumina HiSeq 2000 instrument in 1×101 bp single‐read mode. A total of 92.3 million reads were obtained, corresponding to 99.7× on‐target average sequencing depth based on Agilent SureSelect V2 target region list. Exome sequencing of maternal and paternal DNA samples was performed in‐house (Genomed Ltd.) on Illumina NextSeq 500 instrument in 2×151 bp paired‐end mode to an average depth of 154.1× and 147.5×, respectively. The libraries were prepared and enriched using Illumina Nextera Rapid Capture Exome Kit v1.2; after read alignment, the corresponding target region list was used for sequencing depth calculation. The raw sequencing data have been processed with a custom pipeline based on popular open‐source bioinformatics tools BWA, Samtools, Vcftools, as well as in‐house Perl scripts, using hg19 assembly as a reference sequence. Variant annotations were added by SnpEff/SnpSift software using public databases (dbSNP, ExAC, ClinVar, dbNSFP). After filtering the variants by functional consequence and population frequencies, no suitable candidates were found in a proband among the genes known to date that are responsible for epilepsy and similar disorders, both dominant and recessive. Subsequent removal of variants present in any of the parents allowed identifying a heterozygous *de novo* base substitution (chr6:13283700T>G) corresponding to a missense mutation in *PHACTR1* gene (c.1556T>G / p.Leu519Arg). The cDNA and protein positions in *PHACTR1* throughout the manuscript correspond to transcript NM_030948.3.

Candidate single nucleotide variant was validated using Sanger sequencing using the following primers: *Phactr1‐F2* (5′‐catggtcaacccttctggct‐3′) and *Phactr1‐R2* (5′‐agcagcctttccgcagatta‐3′). Primers covering exon 13 of transcript variant 1 (NM_030948.3) were designed using Primer3Plus. PCR was performed using HsTaq DNA polymerase (Evrogen, Russia) and products were sequenced on an ABI3130xl sequencer (Life Technologies, Carlsbad, CA) using the BigDye Terminator v1.1 Cycle Sequencing Kit (Life Technologies).

A PCR‐restriction fragment length polymorphism‐based screening was performed for population screening of the c.1556T >G mutation using primers *Phactr1‐F2* and *Phactr1‐R2*. The substitution creates a site for *Sfa*NI restriction endonuclease (SibEnzyme, Russia). Restriction fragments were resolved by 3% agarose gel electrophoresis in 1× TBE buffer.

For linkage analysis and parental origin of mutation two nearest heterozygous single nucleotide polymorphisms (SNPs) were chosen according to the WES data: rs3817735 located 5.7 kb upstream and rs202040 5.0 kb downstream of mutation site. SNPs genotypes of the family and informativity were confirmed using Sanger sequencing with primers flanking each SNP locus. To determine phase of mutation, two intersecting fragments 5.7 and 5.0 kb in length were amplified using DNA of proband as a template and strand‐displacement SD Polymerase Hotstart (Bioron, Germany) and primers: *Phactr1‐F1* (5′‐ggagacctgaaaccccatgt‐3′) and *Phactr1‐R2* for rs3817735 containing fragment and *Phactr1‐F2* and *Phactr1‐R3* (5′‐gcctgcgagctctgttatga‐3′) for rs202040 containing fragment. Each fragment covered a SNP on either end and the mutation site on another. Fragments then were TA‐cloned into pTZ57R/T vector (Life Technologies) or pGEM®‐T Easy vector (Promega, Madison, WI) and transformed into *E. coli*, strain XL10‐Gold. Insert‐positive colonies were determined by PCR using primers amplifying SNP as well as mutation sites; plasmid DNA was then isolated and used as a template for PCR to amplify mutation locus and SNP. Mutation phase was determined by Sanger sequencing of PCR products of plasmid DNA amplification using primers: *Phactr1‐F1* and *Phactr1‐R1* (5′‐gggtcatgaagctgagtgga‐3′) for rs3817735, and *Phactr1‐F3* (5′‐gtgcgccttgaagctgat‐3′) and *Phactr1‐R3*. Haplotypes were visualized using open‐source bioinformatics tools, the Haplopainter.^30^

#### 2.3.2 Plasmids

Expression plasmids for Phactr1 wt, Phactr1 RRx, and Phactr1 xxx have been described previously.^24^ L519R mutation was introduced by primer extension site‐directed mutagenesis. In the first round of PCR *Phactr1 Fw*: (5′‐gtcagatcgcaccggtatgaaggactacaaggacga‐3′) and *Phactr1 L519R Rv*: (5′‐tcactgaagcggataCggatcttcctctccc‐3′); *Phactr1 L519R F*w: (5′‐gggagaggaagatccGtatccgcttcagtga‐3′) and *Phactr1 Rv*: (5′‐cgaggaattcggatccttaaggtcgatgaaacctgg‐3′) were used. The two PCR products were then mixed together for a second round PCR with *Phactr1 Fw* and *Phactr1 Rv* primers. The final product was inserted using In‐Fusion cloning kit (Clontech, Mountain View, CA) into a p‐EF‐flag vector for mammalian expression. The expression plasmid for HA‐PP1 plasmid has been described previously.^24^

#### 2.3.3 Cell lines, transfection, and immunofluorescence microscopy

Mouse NIH3T3 fibroblasts were maintained in DMEM with 10% FCS. The 100 000 cells (per well) were plated in a six‐well plate and transfected with expression plasmid using Lipofectamine 2000 (Invitrogen) according to the manufacturer's instructions. Serum‐starved cells were maintained in medium containing 0.3% FCS for 16 h; for serum stimulation, cells were subsequently maintained in 15% FCS for 1 h.

Immunofluorescence microscopy was performed as described earlier.^24^, ^31^ Phactr1 mutants were detected by anti‐FLAG antibody (Sigma F7425). F‐actin was visualized using FITC‐phalloidin (Invitrogen), and nuclei were detected with DAPI.

#### 2.3.4 Proteins and peptides

Actin was isolated from rabbit skeletal muscle essentially as described^32^, ^33^ and saturated with Latrunculin B (Calbiochem) as described previously.^34^

N‐terminally fluorescein isothiocyanate (FITC)‐conjugated peptides were synthesized and HPLC‐purified by the Crick Peptide Chemistry Laboratory: Phactr1 RPEL3 WT (FAM‐eahx‐KREIKRRLTRKLSQRPTVEELRERKILIRFSD‐CONH_2_), Phactr1 RPEL3 L519R (FAM‐eahx‐KREIKRRLTRKLSQRPTVEELRERKI**R**IRFSD‐CONH_2_), Phactr1 RPEL3 R507A (FAM‐eahx‐KREIKRRLTRKLSQ**A**PTVEELRERKILIRFSD‐CONH_2_), Phactr1 RPEL3 R507A L519R (FAM‐eahx‐KREIKRRLTRKLSQ**A**PTVEELRERKI**R**IRFSD‐CONH_2_).

#### 2.3.5 Fluorescence anisotropy

The fluorescence anisotropy assay was performed as described earlier.^34^, ^35^ FITC‐conjugated peptides were used at 0.5 μM while LatB‐actin concentration ranged from 1 nM up to 59 μM. The reactions were set up at room temperature in a total volume of 75 μL and incubated for 2 h in FP binding buffer.

Dissociation constants (*K*_d_) were calculated by nonlinear regression in Prism software using equation: Y=Ab−Af×XKd+X+Af, where *X* is protein concentration, *Y* is total anisotropy, *A*_b_ is anisotropy from bound ligand and *A*_f_ is anisotropy from free ligand.^36^ *K*_d_ values were derived from three independent experiments each in duplicate.

#### 2.3.6 Co‐immunoprecipitation

Cells transfected with Flag‐Phactr1 and HA‐PP1 were lysed in lysis buffer (0.5% Nonidet P‐40, 1 mM EDTA, 50 mM Tris pH 8.0, 120 mM NaCl, 0.1 mM sodium vanadate, protease inhibitors). In 1 mg of total protein was used to immunoprecipitate with anti‐HA‐agarose beads (A2095 SIGMA). After three washes in lysis buffer, proteins were separated by SDS‐PAGE and immunoblotted using anti‐FLAG (Sigma F7425) and PP1α (C‐19, Santa Cruz) antibodies.

## 3 RESULTS

### 3.1 Clinical phenotype

Male‐patient, 1‐year 3‐month‐old was the first child of non‐consanguineous parents with no significant family history. Delivery was at 40 weeks of gestation with a weight of 3540 g and length of 54 cm. Perinatal period was normal. The APGAR score was 8/9. At 2 months of age he gained eye pursuit and social smile but could not achieve head control. The first epileptic spasms in clusters started at 3.5 months and soon became generalized. Video‐electroencephalography (EEG) performed at 7 months of age showed hypsarrhythmia. Brain MRI imaging at 4.5 months revealed hypoplasia of the corpus callosum, cavum veli interposition and non‐obstructive external hydrocephaly above the frontotemporal regions. Delayed myelination was also noted. Repeated study at 6 months of age showed no dynamic changes.

Based on clinical profile and the changes observed in the EEG, multifocal epilepsy with infantile spasms and hypsarrhythmia was diagnosed. Hormone‐therapy with valproic acid were not effective. Other different antiepileptic drugs in different combinations were ineffective for his seizures too. Ketogenic diet and intramuscular adrenocorticotropic hormone injections brought only temporary effect.

### 3.2 *PHACTR1* mutation identified using whole‐exome sequencing

**FIGURE 1:**
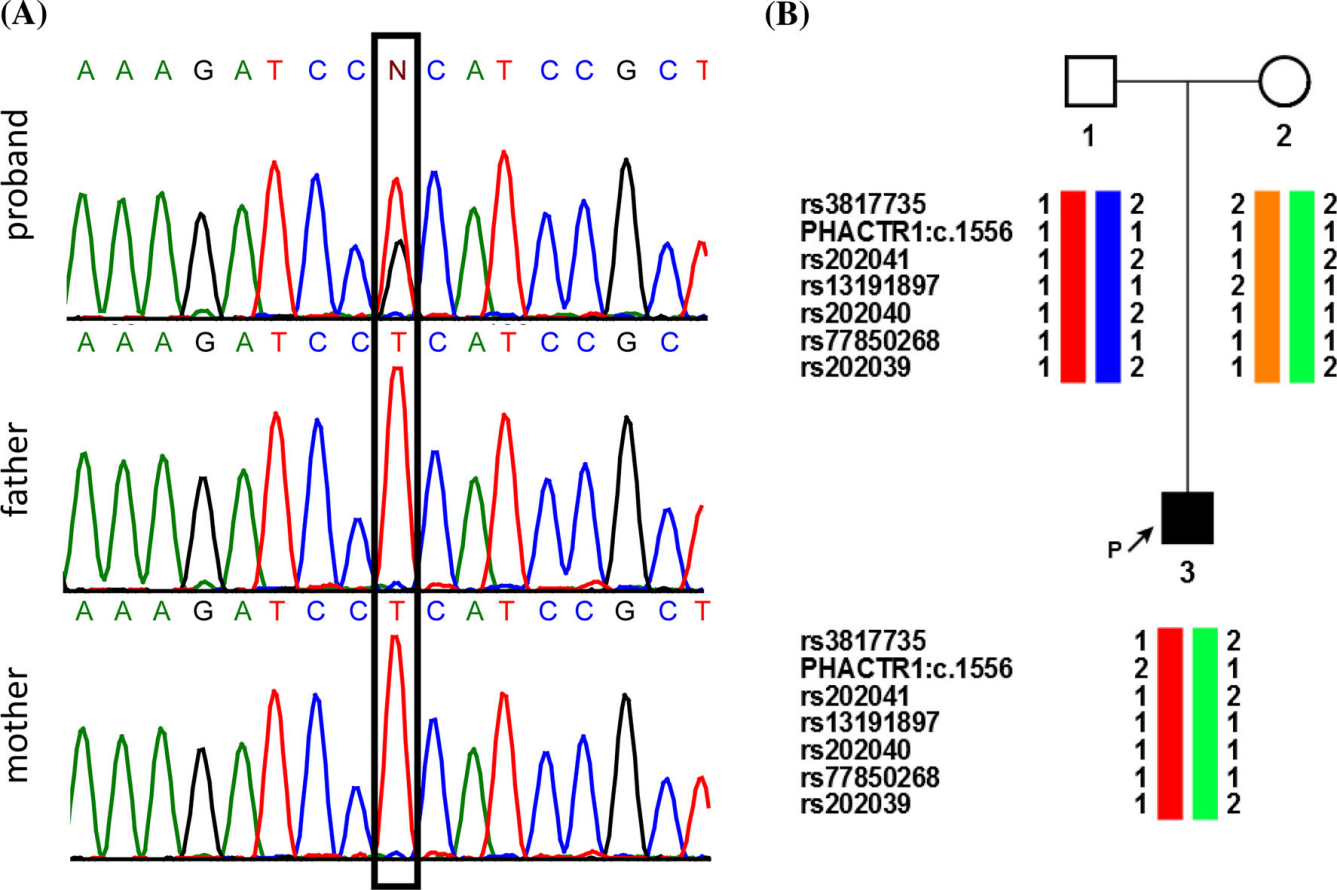
A heterozygous missense mutation (c.1556T>G) in *PHACTR1* in a proband with multifocal epilepsy. (A) Whole‐exome sequencing on a family trio with an affected sibling and unaffected parents identified a heterozygous missense mutation in *PHACTR1* (p.L519R in isoform a). (B) Analysis of linked SNPs identified mutation arose in the paternal allele. Global major alleles were designated as 1 and minor alleles as 2. SNPs, single nucleotide polymorphisms [Colour figure can be viewed at wileyonlinelibrary.com]

Initially, the patient underwent chromosomal microarray analysis on Affymetrix CytoScan HD, which revealed normal male karyotype (ISCN 2016): arr(1‐22)x2,(X,Y)x1. Whole‐exome sequencing was then carried out on a family trio of the affected proband and his unaffected parents. Exome data analysis identified a heterozygous missense mutation c.1556T>G in *PHACTR1* on chromosome 6 (NM_030948.3, p.Leu519Arg, hg19::chr6:g.13283700T>G, Figure 1(A)). This was the only variant present in the affected proband but not in his parents, or in normal population databases including 1000 Genomes, the NHLBI Exome Variant Server, and the GnomAD browser. No other pathogenic mutations were yielded by filtering variants for recessive (homozygous or compound heterozygous) inheritance present in the proband and absent in the unaffected parents. To estimate c.1556T>G allele frequency in the local population, the PCR‐restriction fragment length polymorphism‐based screening for this mutation was carried out in 120 phenotypically normal unrelated individuals from the Central Russian population. No individuals bearing c.1556T>G substitution in either heterozygous or homozygous state were found (data not shown).

Next, we carried out linkage analysis to investigate the parental origin of the mutant allele. The nearest heterozygous SNPs covered by WES data laid on either side of the mutation were: rs3817735 located 5.7 kb upstream, and rs202040 5.0 kb downstream. Downstream of mutation site four additional SNPs were found, and it was possible to determine their phase as well by cloning PCR fragments covering corresponding SNP and substitution in T‐vector with subsequent Sanger sequencing of obtained plasmids. This analysis shows that the mutation occurred *de novo* in paternal allele (Figure 1(B)).

### 3.3 The L519R mutation reduces G‐actin binding affinity, thereby increasing PP1 binding

**FIGURE 2:**
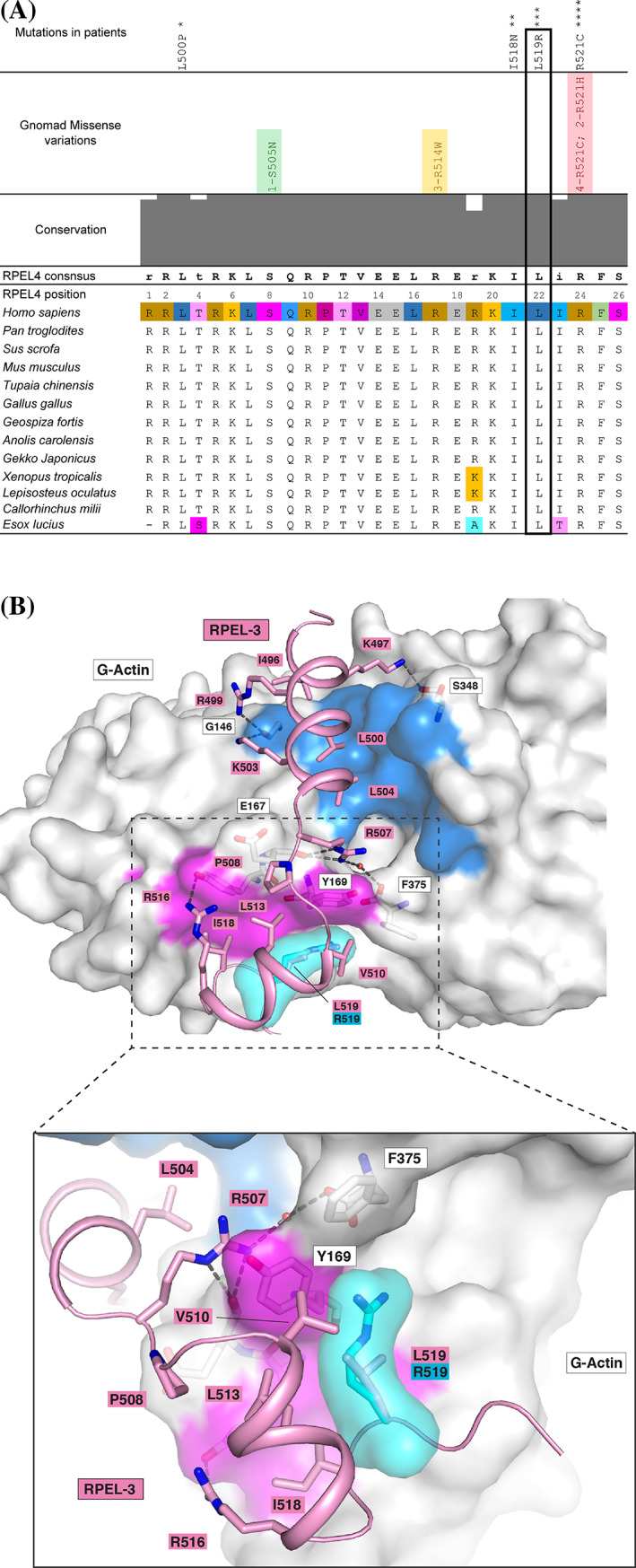
(A) Multiple alignment of PHACTR1 RPEL3 motifs with the highly conserved L519 position boxed. Variations detected in GnomAD database are placed above the conservation plot. Mutations detected in patients with various abnormalities are located above populational variants: * – in Hamada et al., 2018,^29^ ** – in UK DDD study,^37^ *** – in this study, **** – in de Ligt et al., 2012.^38^ (B) The L519R substitution potentially affects interaction with G‐Actin. A ribbon diagram of the PHACTR1 RPEL‐3 (pink) bound to G‐Actin (PDB ID: 4B1Y). RPEL3 Side chains implicated in interaction with Actin are shown as sticks. The mutant arginine‐519 (blue) is simply superimposed on wild‐type leucine‐519, with its surface representation in blue. Although there is no obvious steric clash, the charged character of Arg519 would be expected to weaken the interaction with the hydrophobic ledge [Colour figure can be viewed at wileyonlinelibrary.com]

**FIGURE 3:**
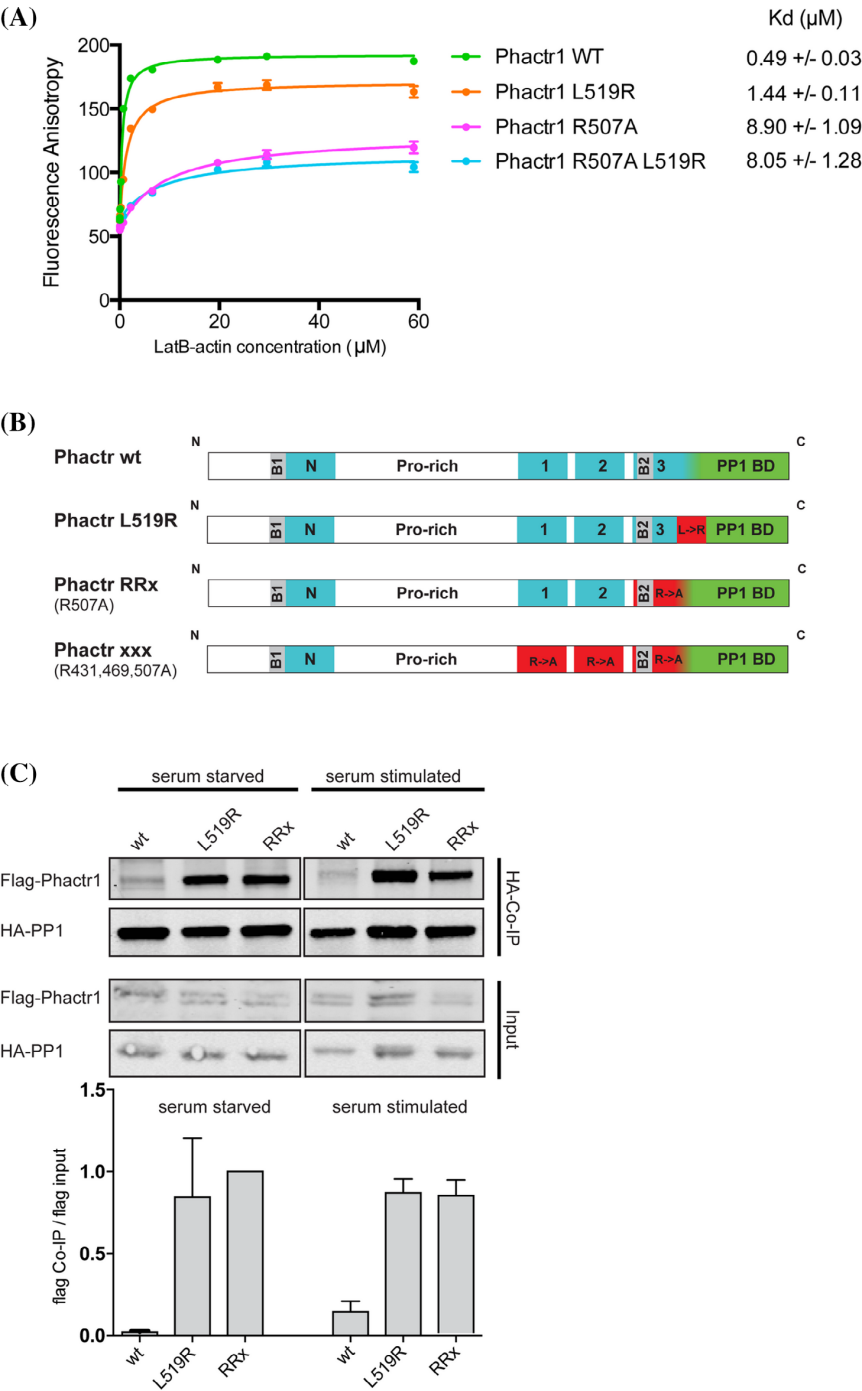
L519R Affects G‐Actin and PP1 binding (A) Fluorescence anisotropy measurement of G‐actin binding to Phactr1 RPEL3 peptides. Anisotropies of FITC‐conjugated 32 aa long peptides (0.5 μM) were measured over a range of LatB‐actin concentrations. Dissociation constants (*K*_d_) are means of three independent experiments ± s.e.m. (B) Domain organization of Phactr1 derivatives tested: wild‐type (wt) L519R, RRx (R507A) and xxx. RPEL motifs are shown in blue, the PP1‐binding domain in green, and nuclear localization signals (B1 and B2) in gray. Mutations are highlighted as red bars. (C) Phactr1 L519R increases binding to PP1 *in vivo*. NIH3T3 cells were transfected with expression plasmids encoding FLAG‐tagged wild‐type PHACTR1, PHACTR1 L519R or PHACTR1 RRx, and HA‐tagged PP1, and maintained in 0.3% serum for 16 hr before addition of 15% serum for 60 utes. Interaction between PP1 and Phactr1 was monitored by testing for recovery of Flag‐Phactr1 in HA‐PP1 immunoprecitates (top), and quantified relative to input (bottom) [Colour figure can be viewed at wileyonlinelibrary.com]

The activity and intracellular location of Phactr1 are regulated by G‐actin, which inhibits importin α‐β binding to nuclear import signals associated with the Phactr1 N‐terminal RPEL motif and C‐terminal RPEL domain, and PP1 binding to its conserved C‐terminal sequences.^23^, ^24^ The L519R mutation lies at a conserved position within RPEL3 (Figure 2(A)).^24^ While there is no obvious steric clash between the more bulky arginine side chain and actin, its charged character would be expected to weaken the interaction with the hydrophobic ledge, potentially reducing G‐actin binding affinity (Figure 2(B)). To test whether the L519R mutation affects G‐actin binding affinity we used the fluorescence polarization anisotropy assay, with FITC‐labeled RPEL3 peptides. The L519R mutation bound G‐actin with affinity 1.44 μM, a 3‐fold reduction compared with the wildtype RPEL3 motif. In contrast, alanine substitution of the R507, the conserved core arginine (R507A) reduced RPEL actin‐binding affinity some 18‐fold, to 8.9 μM, and its combination with L519R had no further effect (Figure 3(A)).

G‐actin binding to PHACTR1 inhibits its interaction with PP1, whose binding site overlaps RPEL3^24^ (R. Fedoryshchak, M. Prechova et al, unpublished data), so we next investigated whether Phactr1 L519R mutation affects PP1 binding. Flag‐Phactr1 derivatives were co‐expressed with HA‐PP1α in NIH3T3 cells, and PHACTR1/PP1 interaction monitored by recovery of Flag‐Phactr1 in HA‐PP1 immunoprecipitates. In this assay, Flag‐PHACTR1 wild‐type interaction with HA‐PP1 was barely detectable in resting cells and increased only slightly upon serum stimulation (Figure 3(B), (C)). In contrast, Flag‐PHACTR1 L519R exhibited greatly increased interaction with PP1, and was comparable with PHACTR1 R507A (“RRx”^24^) in both serum starved and serum‐stimulated conditions (Figure 3(C)). These results are consistent with a model in which the decreased affinity of PHACTR1 L519R for G‐actin decreases its ability to inhibit PP1 binding, thereby potentiating formation of the PHACTR1/PP1 complex (see Discussion).

### 3.4 The Phactr1 L519R mutant exhibits aberrant subcellular localization and function

**FIGURE 4:**
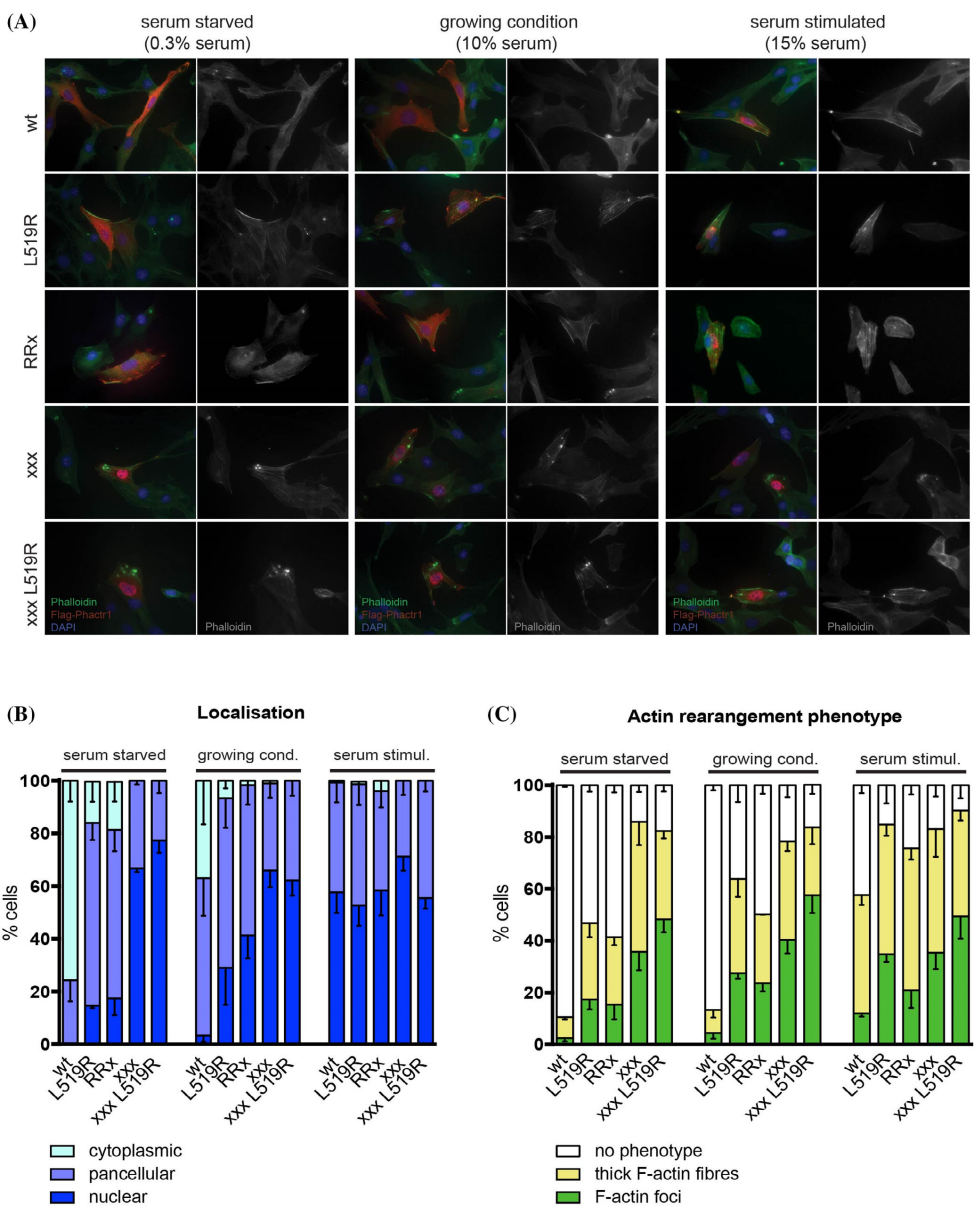
L519R mutation affects Phactr1 localization and function (A) NIH3T3 cells expressing FLAG‐tagged PHACTR1 proteins were either grown in media supplemented with 10% serum or serum starved (by growing in 0.3% serum for 16 h) and then stimulated (by growing in 15% serum for 1 h). Localization of the proteins and F‐actin cytoskeleton was visualized by fluorescence microscopy. (B) PHACTR1 protein localization was scored as cytoplasmic, pancellular or nuclear. Error bars represent the s.e.m. of three independent experiments. (C) Quantification of cytoskeletal phenotypes in cells expressing PHACTR1 wt, L519R, RRx, xxx and xxxL519R. Error bars represent the s.e.m. of three independent experiments [Colour figure can be viewed at wileyonlinelibrary.com]

We previously showed that in NIH3T3 fibroblasts, G‐actin binding is critical for regulation of PHACTR1 subcellular localisation and for PP1‐dependent induction of cytoskeletal rearrangements.^24^ We therefore next examined the effect of L519R mutation on PHACTR1 localisation. In NIH3T3 fibroblasts, serum stimulation induces PHACTR1 nuclear accumulation, and the integrity of the C‐terminal RPEL motifs, including RPEL3, is required to maintain its cytoplasmic localisation in resting cells.^24^ While wildtype PHACTR1 was predominantly cytoplasmic in resting cells, PHACTR1 L519R exhibited predominantly pancellular or nuclear localization, behaving in a similar fashion to the PHACTR1 R507A; PHACTR1xxx, that carries alanine substitutions at the core ariginines of RPEL1, RPEL2 and RPEL3, was constitutively nuclear (Figure 4(A), (B)).

We next examined the effect of the L519R mutation on PHACTR1‐induced F‐actin assembly in NIH3T3 cells. Similar but less pronounced cytoskeletal rearrangements were also seen upon serum stimulation of cells overexpressing wildtype PHACTR1. Overexpression of PHACTR1 L519R was sufficient to induce thick F‐actin fibers and F‐actin foci in resting or exponentially growing cells, which were increased upon serum stimulation and expression of PHACTR1 R507A had a similar effect (Figure 4(A), (C)). In contrast, expression of wildtype PHACTR1 weakly induced F‐actin rearrangements only upon serum stimulation, and as previously reported PHACTR1xxx was more active, with the majority of the cells exhibiting F‐actin rearrangements (Figure 4(A), (C)). Taken together with the results in the preceding section, these data suggest that the PHACTR1 L519R mutation is a gain‐of‐function mutant, which results in both altered PHACTR1 subcellular localization and increased PP1‐dependent downstream signaling.

## 4 DISCUSSION

Here we describe a *de novo* mutation, L519R, in *PHACTR1* in a sporadic case of multifocal epilepsy with infantile spasms and hypsarrhythmia. The Phactr family of G‐actin binding proteins are PP1 cofactors are implicated in the regulation of cytoskeletal dynamics.^24^, ^39^, ^40^ Family members have previously been associated with coronary artery disease, Parkinson's disease, melanoma and lung cancer in genetic studies.^39^, ^41^, ^42^, ^43^, ^44^ Our functional analysis indicates that the L519R mutation effectively results in activation of PHACTR1 by potentiating its ability to bind PP1 (see Graphical Abstract).

The PHACTR proteins each contain four RPEL motifs: one at the N‐terminus, and three within the C‐terminal RPEL domain. The most C‐terminal motif, RPEL3, overlaps the sequences required for interaction with PP1. Our *in vitro* G‐actin binding experiments indicate that the L519R mutation reduces the RPEL3 affinity some 3‐fold. In contrast, mutation of the core RPEL3 arginine, R507A, reduces affinity 18‐fold. Nevertheless, in co‐immunoprecipitation experiments we found that PHACTR1 L519R exhibited greatly increased association with PP1, to an extent comparable to the R507A mutant. We suggest that G‐actin concentrations in the expression assays are such that the relatively small decrease in binding affinity is sufficient to tip the balance of interaction in favor the formation of PHACTR1/PP1 complex. In fibroblasts, over‐expression of PHACTR1 RPEL mutants that exhibit defective actin binding induces assembly of thick F‐actin fibers and F‐actin foci, and this requires PP1 binding.^24^ We found that the L519R mutant also exhibited elevated levels of F‐actin remodeling, comparable to those seen with the R507A mutant. This result is consistent with the increased ability of L519R to interact with PP1.

In addition to its effects on PP1 interaction and cytoskeletal rearrangement, G‐actin binding also affects the subcellular localization of PHACTR1. In transfected fibroblasts, PHACTR1 is predominantly localized in the cytoplasm, because binding of importin αβ to NLS associated with RPEL motifs, is prevented by G‐actin binding.^24^ Analysis of core arginine mutations at individual RPEL motifs showed that RPEL3, R507A, had the biggest effect on PHACTR1 subcellular localization.^24^ In line with the effects seen in the PP1 binding analysis, PHACTR1 L519R exhibited increased but not exclusive nuclear localization, similar to the R507A mutant. Although the relevance of PHACTR1 subcellular localization to its function remains unclear, this observation raises the possibility that its effects may be mediated by nuclear targets of PHACTR1/PP1. Summing up, L519R variant is classified as pathogenic (II) with evidence criteria PM2, PP3, PS3, PS2 according to the ACMG standards.^45^

Subcellular imaging in cultured hippocampal neurons detected PHACTR1 distributed throughout cell bodies and dendrites, with apparent enrichment in dendritic spines, and in the perinuclear region, but not in cell nuclei.^18^ PP1α and PP1γ1 labeling are enriched in spines: PP1γ1 is highly and specifically concentrated in the postsynaptic density (PSD), while PP1α is enriched in and around the PSD.^46^ Given the effects of the L519R mutation on PHACTR1 subcellular localization as well as its interaction with PP1, it is therefore possible that its phenotype reflects redistribution of PP1.

The PHACTR1 L519R mutation acts positively to increase formation of the PHACTR1PP1 complex, and therefore stands in contrast to the mouse *humdy* mutant, which carries a mutation that prevents formation of the Phactr4/PP1 complex.^20^, ^21^ *humdy* exhibits failure to close the neural tube and optic fissure in the early development of the central nervous system (CNS), causing exencephaly and retinal coloboma, and is associated with cytoskeletal and cell cycle defects.^20^, ^21^ Although *humdy* acts as an incompletely penetrant dominant allele, its phenotypes are likely reflect its action as a dominant negative that prevents recruitment of PP1 to Phactr4 binding sites.^20^

A recent study demonstrated the *de novo PHACTR1* mutations to be the cause of West syndrome in two Japanese patients.^29^ The authors used mouse models to demonstrate that these mutations act dominantly. Phactr1 knockdown causes defects in cortical neuron migration during corticogenesis, which were rescued by an RNAi‐resistant PHACTR1 but not by the four mutants (L500P, N479I, I518N, R521C). In this assay, co‐expression of the mutants, interfered with rescue by RNAi‐resistant PHACTR1, indicating a dominant negative effect of all the mutant alleles. In contrast, only the R521C mutant was determined to have a dominant negative effects on dendritic development *in vivo*, the three other mutants appearing to be degraded under the experimental conditions used. Electrophysiological analyses revealed abnormal synaptic properties in Phactr1‐deficient excitatory cortical neurons.

Recently it was shown that PHACTR1 interacts with Slack (K_Na_1.1), a Na^+^‐activated K^+^ channel, and dissociates from it upon channel activation. Mutations in Slack are associated with malignant migrating partial seizures of infancy (MMPSI), characterized by infantile seizures and intellectual disability.^28^ Interestingly, MMPSI mutations not only constitutively activate the Slack channel, but also affect its interaction with PHACTR1.^27^ Although it remains unclear whether MMPSI mutations prevent PHACTR1 from binding Slack at all, or only block its release upon channel activation, it is tempting to speculate that the increased basal level of PHACTR‐PP1 activity resulting from the L519R mutation might effectively mimic the Slack MMPSI mutations. For example, it could result in decreased levels of inhibitory phosphorylations on Slack, or in its vicinity, or increase the activity of free PHACTR1/PP1 against downstream neuronal PHACTR1 substrates. Nevertheless, recent data^47^ have confirmed that Phactr1 regulates Slack (KCNT1) channels via protein phosphatase 1 (PP1). Phactr1 is shown to be required to link the channels to actin. Co‐expression of Phactr1 with wild‐type Slack channels reduces the current amplitude. Furthermore, a Phactr1 *humdy* mutation (R536P) that disrupts the binding of PP1 but not that of actin fails to alter Slack currents.

We compared the clinical picture of the patients with *PHACTR1* mutations with those patients bearing variants of *KCNT1* (encoding the Slack channel). It demonstrates a significant degree of similarity (Table 1). The variant c.1561C>T, R521C is found in a patient with mental retardation, seizures (started 3 weeks after birth), severely delayed psychomotor development, spastic tetraparesis, joint contractures and scoliosis.^38^ The full‐scaled clinical picture resembles our case in its initial development. Nevertheless, p.R521C variant was identified four times in heterozygous state in GnomAD database with additional two individuals bearing p.R521H substitution (Figure 2(A)). While these data emphasize the detrimental effect of changes at PHACTR1 amino‐acid 521, further work is needed to establish the molecular basis of the connection between the p.R521C mutation and epilepsy.

**TABLE 1:** Comparison of clinical picture of patients with *KCNT1* mutations with those who have *PHACTR1*

N; m/f	Age	Gene	Mutation	Age at seizure onset	Seizure type at onset	Neurological evaluation	MRI (age)	Current AEDs	AE trials
1 m	10y	KCNT1	c.2800G>A, p.Ala934Thr	1 m	Focal motor (clonic) with secondary generalization	Axial hypotonia, Microcephaly, Preserved eye contact, No language, No walking	Myelination delay, Cortical Atrophy (10y)	VGB, TPM, LVT	VPA, CLB, CBZ, VGB, CZP, KD, TPM
2 m	10y	KCNT1	c.1283G>A, p.Arg428Gln	2 m	Focal motor	Axial hypotonia, Microcephaly, Preserved eye contact, No language, No walking	Myelination delay, Thin corpus callosum, Cortical atrophy (5y)	PB, VGB, CZP, TPM, KD	PB, PHT, VGB, CZP, TPM, KD
3 m	8y	KCNT1	c.1283G>A, p.Arg428Gln	17 h	Focal and generalized tonic, autonomic seizures (cyanosis, bradycardia)	Axial hypotonia, Microcephaly, Pyramidal syndrome, Lack of contact	Myelination delay, Thin corpus callosum, Cortical atrophy (4,5y)	CZP, TPM, LVT	PB, PHT, VPA, CBZ, CZP, VGB, KD, TPM, LTG
4 m	0.5y	KCNT1	c.1283G>A, p.Arg428Gln	2 h	Focal motor with secondary generalization	Axial hypotonia, Microcephaly, Lack of contact	Thin corpus callosum (2 m)	VGB, CZP, VPA, TPM	PB, PHT, CZP, LVT, VGB, VPA, TPM
5 m	0.5y	KCNT1	c.1421G>A, p.Arg474His	2 w	Focal motor	Axial hypotonia, Lack of contact	Normal (1 m)	VGB, TPM, VPA	CZP, PHT, PB, LVT, VGB, TPM, VPA
6 f	0.5y	KCNT1	c.2280C>G, p.Ile760Met	3d	Focal motor and autonomic (cyanosis)	Axial hypotonia, Lack of contact	Normal (1 m and 2 m)	CBZ, STP, CZP, LTG	VPA, PHT, VGB, HC, CBZ, STP, CZP, LTG
7 f	1.5y	PHACTR1	c.1499T>C, p.Leu500Pro	3 m	Focal seizures with tonic component	Generalized hypotonia	Progressive atrophy, Myelination delay	VPA, LTG, LVT, ACTH	VPA, LTG, LVT
8 m	3y	PHACTR1	c.1436A>T, p.Asn479Ile	3 m	Epileptic spasms in cluster	walked alone at 18 months, peak, single words at 24 m, autism spectrum disorder	Normal (3 m and 3 y)	ACTH, VPA	
9 m	1y3 m	PHACTR1	c.1556T>G, p.Leu519Arg	3.5 m	Focal motor with secondary generalization	Generalized hypotonia	Hypoplasia of the corpus callosum cavum veli interpositi non‐obstructive external hydrocephaly	VPA, OXC	VPA, steroids, LVT, VGB, KD, ESM, ACTH, ZNS, OXC

Abbreviations: ACTH, adrenocorticotrophic hormone; ADHD, attention‐deficit hyperkinetic disorder; AED, anti‐epileptic drug; CBZ, carbamazepine; CLB, clobazam; CZP, clonazepam; ESM, ethosuximide; LTG, lamotrigine; LVT, levetiracetam; NA, not available; OXC, oxcarbazepine; PHT, phenytoin; PLP, pyridoxal phosphate; STP, stiripentol; TPM, topiramate; VGB, vigabatrin; VPA, valproic acid; ZNS, Zonisamide.

In conclusion, we have identified an activating mutation, L519R, in the PHACTR1 gene in a sporadic case of multifocal epilepsy. By altering PHACTR1's ability to bind G‐actin, L519R effectively activates the PHACTR1/PP1 complex, leading to enhanced dephosphorylation of target substrates. G‐actin binding also controls PHACTR1 subcellular localization, and the L519R mutation could also exert its effects through changes in PHACTR1/PP1 substrate targeting. Our findings suggest that actin treadmilling and PHACTR1 is involved in regulation of the phosphorylation/dephosphorylation balance in synapses and that activation of PHACTR1/PP1 is involved in epileptogenesis. This could be used for etiotropic therapy opportunity as well as for genetic counseling of the affected family. Our study adds PHACTR1 to the list of epilepsy‐related genes and contributes to the diverse molecular mechanisms of the disease.

## CONFLICT OF INTEREST

All authors declare no conflict of interests.

### PEER REVIEW

The peer review history for this article is available at https://publons.com/publon/10.1111/cge.13926.

## Data Availability

The data that support the findings of this study are available on request from the corresponding author. The data are not publicly available due to privacy or ethical restrictions.
